# Vagal Afferent Mediates the Anorectic Effect of Peripheral Secretin

**DOI:** 10.1371/journal.pone.0064859

**Published:** 2013-05-30

**Authors:** Jessica Y. S. Chu, Carrie Y. Y. Cheng, Revathi Sekar, Billy K. C. Chow

**Affiliations:** 1 School of Biological Sciences, The University of Hong Kong, Pokfulam, Hong Kong SAR, China; University of Hong Kong, Hong Kong

## Abstract

Secretin (SCT) is a classical peptide hormone that is synthesized and released from the gastrointestinal tract after a meal. We have previously shown that it acts both as a central and peripheral anorectic peptide, and that its central effect is mediated via melanocortin system. As peripheral satiety signals from the gastrointestinal tract can be sent to the brain via the vagal afferent or by crossing the blood-brain barrier (BBB), we therefore sought to investigate the pathway by which peripheral SCT reduces appetite in this study. It is found that bilateral subdiaphragmatic vagotomy and treatment of capsaicin, an excitotoxin for primary afferent neurons, could both block the anorectic effect of peripherally injected SCT. These treatments are found to be capable of blunting i.p. SCT-induced Fos activation in pro-opiomelanocortin (POMC) neurons within the hypothalamic Arcuate Nucleus (Arc). Moreover, we have also found that bilateral midbrain transaction could block feeding reduction by peripheral SCT. Taken together, we conclude that the satiety signals of peripheral SCT released from the gastrointestinal tract are sent via the vagus nerves to the brainstem and subsequently Arc, where it controls central expression of other regulatory peptides to regulate food intake.

## Introduction

SCT, a 27-amino acid peptide hormone primarily produced from duodenal S cells, belongs to the vasoactive intestinal polypeptide (VIP)/secretin/glucagon/growth hormone-releasing hormone (GHRH) peptide family [1]. The physiological role of this gastrointestinal hormone is to provide an optimum condition for food digestion in the intestine by regulating gastric secretion and emptying [2]–[5] and stimulating the release of bicarbonate-enriched fluid from the pancreas [6], [7] and bile from the liver [8], [9]. Plasma SCT levels increase postprandially [10]–[13], suggesting that SCT may function as a peripheral satiety signals. We have recently identified SCT and its receptor expression in the hypothalamus [14], [15], which is a region implicated in energy homeostasis, and that SCT is an anorectic peptide, which inhibits food intake in mice when administered peripherally or centrally [16]. SCT could also activate POMC neurons by augmenting POMC mRNA levels in the Arc, suggesting a role for Arc POMC in mediating the anorectic action of SCT.

Vagal afferent fibres of the vagus nerve are the major neuroanatomical structure connecting the alimentary tract to the nucleus of the solitary tract (NTS) in the hindbrain. Gut hormones transmit satiety signals to the brain via the vagal afferent pathways, bloodstream or both. SCT is capable of crossing the BBB and reach the brain by transmembrane diffusion via a non-saturable mechanism [17] and SCTR transcript and protein are detected in the Arc and PVN [16], suggesting that circulating SCT may directly activate neurons in hypothalamic centres for appetite control. However, SCTR was found in vagal afferents, and also subdiaphragmatic vagotomy was able to block i.p.-SCT-induced Fos expression in the rat brain [18], suggesting an alternative pathway that peripheral SCT may communicate with the central feeding centre via vagus nerve. In this study, we investigated the potential role of vagus nerve in relaying SCT-induced signal for satiety to the brain. We studied the effect of peripherally administered SCT on food intake in mice with subdiaphragmatic vagotomy and capsaicin, a specific afferent neurotoxin, treatment. We also examined the effect of these procedures on SCT-induced Fos expression in POMC neurons in the Arc.

## Materials and Methods

### Animal Handling

The procedures of animal care and handling were in accordance with the protocol approved by the Committee on the Use of Live Animals in Teaching and Research (CULATR) of the University of Hong Kong. All experiments were carried out using wild-type adult male mice (20–25 g), which were kept in a temperature-controlled room with a 12-h light/dark cycle. Mice were fed ad libitum with standard rodent chow (no. 5010, Test Diet, IN) and water, unless otherwise stated, and for those that were subjected to surgical experiment, subcutaneous burenorphine (0.1 mg/kg, q12 h) and amoxicillin (100 mg/kg, 12–24 hourly) were administered for the 5 days of recovery time as analgesia and anti-flammatory drug.

### Subdiaphragmatic Vagotomy

Mice were anesthetized and surgery was conducted in aseptic conditions. The stomach and lower oesophagus were exposed after an upper midline laparotomy. The stomach was gently retracted, and the anterior and posterior vagal trunks were exposed and transected. In sham-operated mice, a similar abdominal incision was made; the vagal trunks were identified but not transected. All operated mice were allowed to recover 2 weeks prior to ICV experimentation.

### Capsaicin Treatment

Mice were treated with increasing doses of capsaicin in one daily intra-peritoneal (i.p.) injections over 3 consecutive days (8 mg/kg, 20 mg/kg and 30 mg/kg). Capsaicin was dissolved in 10% ethanol and 10% Tween 80 in 0.9% sterile saline. To counteract respiratory impairment associated with capsaicin administration, mice were pretreated with atropine (0.2 mg/kg), terbutaline (0.2 mg/kg), and aminophylline (20 mg/kg). Control mice underwent the same experimental procedure described above, but were injected with vehicle without capsaicin. Feeding studies were performed 2 weeks after the last capsaicin injection. To check the effectiveness of capsaicin pretreatment, the abdominal constrictions in response to i.p injection of 0.01% acetic acid were evaluated. Mice that didn’t display abdominal constrictions to the injection had the vagal afferent successfully lesioned.

### Bilateral Midbrain Transection

Bilateral midbrain transection was performed at a site rostral to the NTS to investigate whether the ascending efferents of the NTS are necessary in mediating i.p. SCT effects on food intake. Briefly, the head of the mouse was fixed in a stereotaxic instrument. A steel knife (1 mm wide) was lowered into the brain in a coronal plane, bilaterally 0.5 mm from the midline, 3.5 mm caudal to the bregma, and 4.5 mm ventral to the dura. For sham operation, the skull was exposed and two holes were drilled on both sides of the midline, while the brain was left intact. After the feeding test, the brains were removed and the exact location of the lesion was verified histologically.

### Intracerebroventricular (i.c.v.) Cannulation and Injection

Mice were implanted with a permanent 11-mm-long, 21-gauge stainless steel cannula projecting to the lateral ventricle, according to the co-ordinates of Paxinos and Franklin (2001). (Coordinates relative to bregma were as follows: i.c.v, 1.00 mm lateral, 0.5 mm posterior, and 2.0 mm ventral). Cannula placement was confirmed by injection of a dye and injections were done using PE-10 tubing attached to an injector and a 10-µl Hamilton syringe. All animals were allowed to recover from surgery for a minimum of 5–7 days, then artificial cerebrospinal fluid (aCSF; prepared according to Alzet protocol, 5 µl) and SCT (454 ng/5 µl; 60677; AnaSpec, San Jose, CA) were injected into the lateral ventricle.

### Feeding Studies

Experimental mice (sham-operated, vagotomized and capsaicin-treated) were fasted for 18 h before study. During the early light phase (10∶00), mice were injected with SCT (i.c.v.: 0.15 nmol; i.p.: 5 nmol), whereas control mice were treated either i.c.v.-aCSF or i.p.-PBS. After injection, mice were individually put in metabolic cages provided with preweighted amount of chow. Cumulative food intakes were measured at specific time points after injection.

### Immunohistochemistry

Immunohistochemical staining was performed on paraffin-embedded brain sections (7 µm). Briefly, sections were deparaffinizated, rehydrated, and permeabilized in PBS-BT (phosphate-buffered saline supplemented with 2% bovine serum albumin and 0.5% Triton X-100). Thereafter, endogenous peroxidase activity was blocked by treatment with 3% hydrogen peroxide, followed by microwave antigen retrieval. After blocking with normal serum, sections were incubated with primary antibody, and immunoreactive signals were detected using either the Vectastain ABC Elite kit (PK-6101, Vector Laboratories, Burlingame, CA), or proceed to fluorescence secondary antibody incubation for visualization using Zeiss LSM 510 Meta computerized image analysis system. The number of positive immunoreactive cells was counted from both sides of the brain. The sum of the number of Fos-expressing cells on both sides was calculated in each animal and used for statistical analysis. Primary antibodies used were: 1∶500 rabbit anti-Fos Ab (Santa Cruz Biotechnology, Santa Cruz, CA) and 1∶5000 goat anti-POMC Ab (Abcam, Cambridge, MA; Raised against synthetic peptide C-NAIIKNAYKKGE, corresponding to C terminal amino acids 256–267 of Human POMC). Secondary antibodies used were: 1∶300 Alexa Fluor 488 donkey anti-rabbit IgG (Invitrogen, Carlsbad, CA) and 1∶300 Alexa Fluor 555 donkey anti-goat IgG (Invitrogen).

### Statistical Analysis

All data are shown as means ± SEM and were analyzed by two-way ANOVA with Bonferroni posttests using the computer software PRISM (version 4.0; GraphPad).

## Results

### Peripheral SCT Decrease Food Intake via the Vagal Afferent Nerve

**Figure 1:**
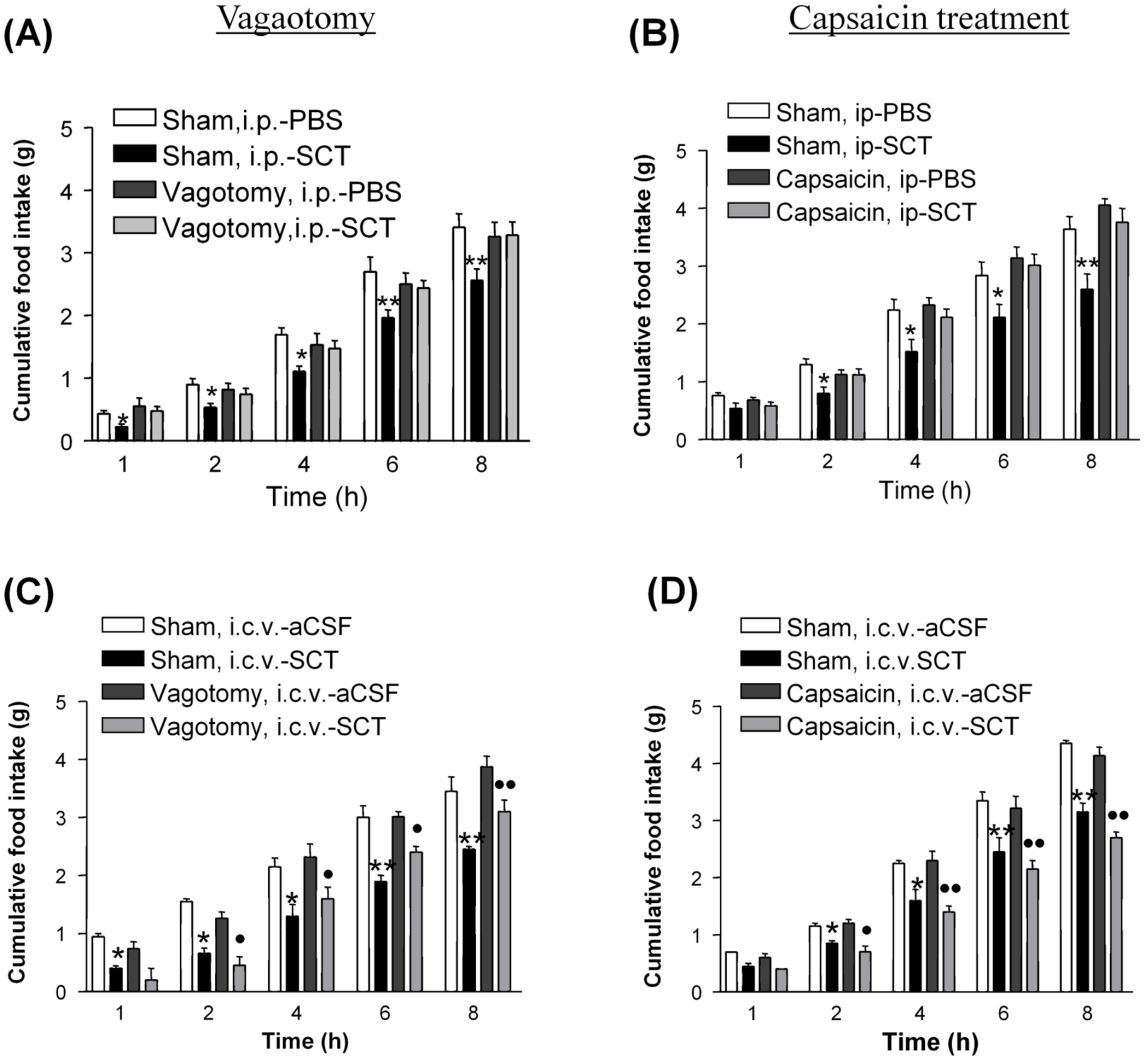
Effect of vagotomy (A,C) and capsaicin treatment (B,D) on i.p. 5 nmol SCT (A,B)- and i.c.v. 0.15 nmol SCT (C,D)-induced food intake. Food intake amount were represented as cumulative value at different time points (1, 2, 4, 6, and 8 h) after SCT treatment. Data are expressed as the means ± SEM (n = 10/group). * p<0.05, ** p<0.01 compared with the sham-operated mice injected with i.p.-PBS/i.c.v.-aCSF. • p<0.05, •• p<0.01 compared with surgical/chemical-treated mice injected with i.p.-PBS/i.c.v.-aCSF.

We have previously demonstrated that both centrally injected and peripherally administered SCT decreases food intake in mice, and that the anorectic effect of centrally injected SCT is mediated by the melanocortin system [16]. To investigate the pathway through which peripherally injected SCT reduces food intake, the effect of vagotomy on SCT-injected and control mice was studied. A single i.p. administration of SCT (5 nmol) was shown to significantly decrease food intake in sham-operated mice, but this inhibitory effect on food intake was abolished in vagotomized mice (Figure 1A). Since the subdiaphragmatic vagus is composed of a number of branches, including the thick myelinated fibers, which carry information mainly from the mechanoreceptors of the heart and blood vessels [19], and a number of efferent and afferent signals to and from a variety of abdominal organs, a specific afferent neurotoxin, capsaicin, that kills only the unmyelinated fibers, was used to further characterize if vagal afferent is involved in the inhibition of food intake by SCT. Vehicle-treated mice exhibited significant suppression of food intake after i.p. SCT (5 nmol); whereas in capsaicin-treated mice, i.p. SCT failed to suppress feeding (Figure 1B). This suggested that unmyelinated fibers in the vagus nerve are responsible for carrying the signals from i.p.-injected SCT to the higher center. Furthermore, as vagotomy and capsaicin treatment were only effective in abolishing the effect of peripherally administered SCT but not centrally injected SCT (Figure 1C, 1D), therefore, these data suggest that the vagal afferent is essential in mediating the anorectic action induced by peripherally SCT.

### Peripheral SCT Activates POMC Neurons in the Arc via the Vagal Afferent Nerve

**Figure 2:**
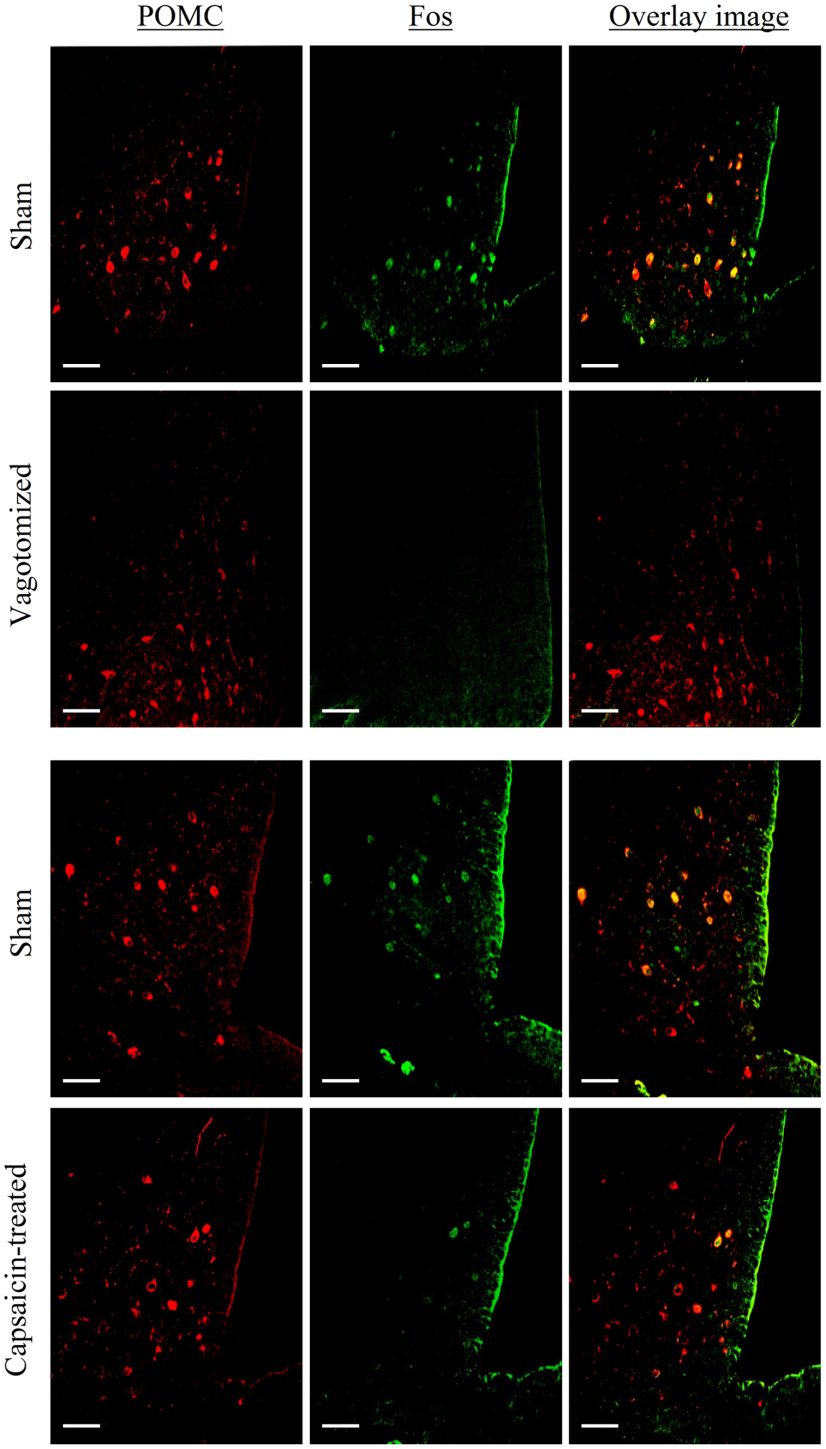
The effect of vagotomy, capsaicin treatment, and sham operation on SCT-induced Fos expression in POMC neurons. Bars, 80 µm.

We have previously shown that peripherally injected SCT elevates POMC and Fos expression in POMC neurons in the Arc [16], suggesting peripheral SCT modulates food intake in part via the POMC neurons. To study if this signal is sent via the vagus nerve to Arc, vagotomy and capsaicin treatment were again used. In POMC neurons of sham-operated mice, i.p.-SCT could consistently induce Fos expression, whereas in both capsaicin-treated and vagotomized mice, i.p.-SCT-induced Fos expression in POMC neurons was reduced (Figure 2), suggesting that SCT signals its satiety effects to the higher centre through the vagus nerve.

### Peripheral SCT Causes Neuronal Activation in the Brainstem

**Figure 3:**
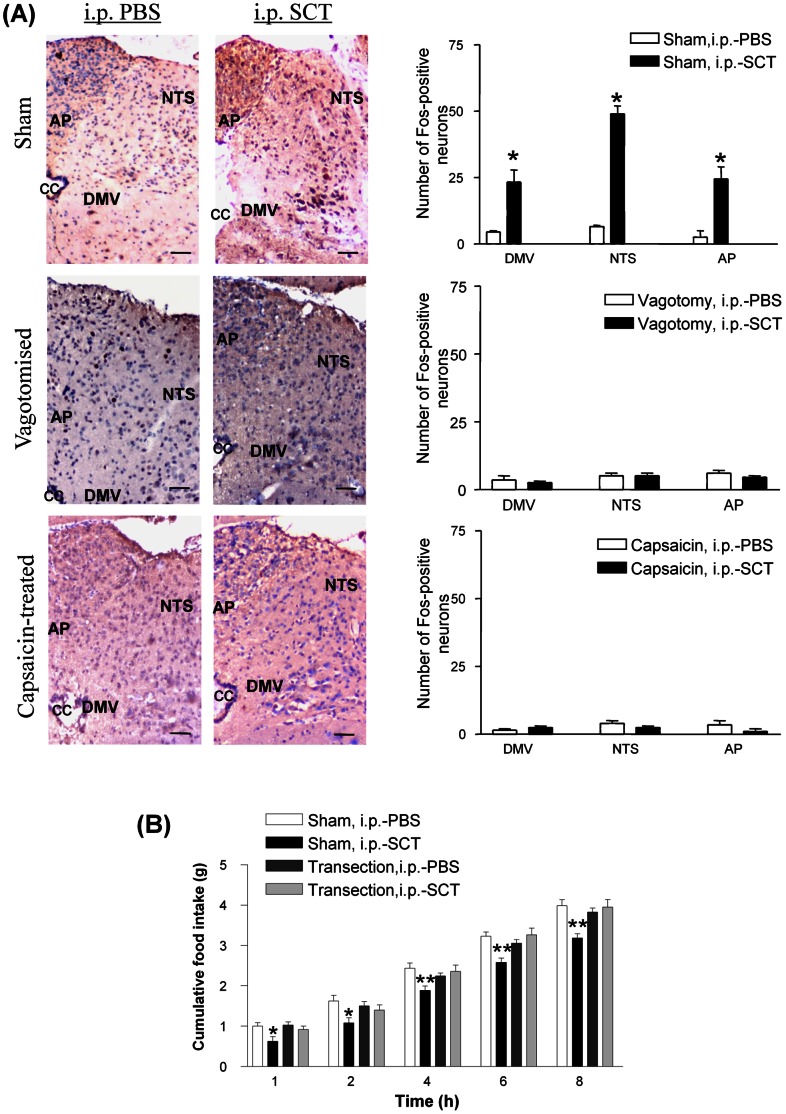
SCT transmits satiety signals to the higher brain in part via the vagal afferent pathway through the brainstem. (A) Changes in Fos immunoreactivity in the AP, NTS and DMV upon i.p.-SCT in sham-operated, vagotomized and capsaicin-treated mice. Left panel: Immunohistochemical staining showing changes in Fos expression. Right panel: Bar chart showing the number of Fos immune-positive cells in the respective regions. Data are expressed as the means ± SEM (n = 5/group). *p<0.05, compared with the control with the respective controls with saline injection. (B) Cumulative food intake at different time points (1, 2, 4, 6, and 8 h) after i.p.-SCT (5 nmol) or i.p.-PBS (vehicle) in 18 h-fasted mice with bilateral midbrain transection or sham operation. Data are expressed as the means ± SEM (n = 10/group). *p<0.05, **p<0.01 compared with the sham-operated mice with i.p.-PBS injection.

The brainstem and the hypothalamus are reciprocally connected. Within the brainstem, the dorsal vagal complex (DVC), consisting of the area postrema (AP), NTS, and the dorsal motor nucleus of the vagus nerve (DMV), is crucial for interpretating and relaying of peripheral signals including the vagal afferents to the hypothalamus. To determine if the anorectic effect of peripheral SCT is sent via the brainstem to the Arc, Fos expression, a marker for neuronal activation, was monitored by immunohistochemical examination after peripheral injection of SCT. We found that i.p.-SCT could increase the number of Fos-positive cells in the AP, NTS and DMV, whereas subdiaphragmatic vagotomy significantly decreased the number of i.p.-SCT-induced Fos positive cells in these areas (Figure 3A). This data is consistent with the previous finding that showed a dose-dependent increase in the number of Fos-positive neurons in AP, NTS, and DMV [18], and indicated that the vagus nerve is responsible for relaying the peripheral SCT anorectic signal to the central nervous system via the brainstem. To further substantiate the role of vagus pathway in mediating i.p.-SCT effect on food intake, midbrain transection was performed at a site rostral to the NTS to cause lesion in areas containing the ascending efferents of the NTS. Our data showed that transection of all ascending NTS projection could block the ability of i.p.-SCT in reducing food intake (Figure 3B), supporting that neural pathway ascending from the NTS play a role in the transmission of SCT-induced anorectic signals to the hypothalamus, and that the route for i.p.-SCT induced anorectic behaviour is gut vagus nerve NTS forebrain regions.

## Discussion

The hormone SCT is released from intestinal enteroendocrine S-cells after a meal, and its action in regulating pancreatic exocrine secretion of water and biocarbonate, gastric acid secretion, and gastric motility are well studied. Recently, our laboratory has demonstrated that the peptide, when injected peripherally, could also act as a satiety signal, decreasing food intake and altering the expression of neuropeptides within the Arc [16]. In this study, we further substantiate this finding and demonstrate the role of the vagus nerve, particularly the non-myelinated fibers in the afferent pathway, in mediating SCT’s effect on appetite. We found that vagus nerve pathway is specifically employed by peripheral SCT, but not centrally expressing SCT in regulating the feeding behavior, as bilateral subdiaphragmatic vagotomy and capsaicin treatment could only block the anorectic effects, as well as the induced activation of POMC neurons in the Arc, triggered by i.p.-SCT, but not i.c.v.-SCT. This finding clearly indicates that vagal afferents are required for the action of circulating SCT. Supporting this notion, SCT was shown to regulate gastric relaxation [20], pancreatic secretion [21], as well as gastric acid secretion [22] via the vagal afferent pathways originating in the gastrointestinal mucosa. In addition, its receptor was previously found to be expressed in the nodose ganglion of the vagus nerve [18], and is axonally transported and accumulated in vagus nerve fibers that terminate in the forestomach [23], [24]. All these findings provide anatomical evidence to support the functional role of SCT in modulating appetite via the vagus nerves.

The pathways through which vagus nerves transmit SCT-induced pancreatic exocrine secretion of fluid and bicarbonate, gastric acid secretion, and gastric motility have previously been suggested [20]–[22]. The effect was believed to be mediated by NTS, a region within the brainstem integrating and relaying information between the gut via the vagus nerve and other second order neurons in the higher centre, including Arc, dorsomedial and PVN [25] in the hypothalamus. Previous report suggested that i.p.-SCT could induce Fos expression in the NTS and Arc through the vagal pathway [18], and depolarize NTS neurons through activation of a nonselective cationic conductance [26]. Similarly, we now show a direct neuronal activating effect of i.p.-SCT on AP, NTS and DMV in sham-operated mice but not in vagotomised and capsaicin-treated mice. In addition, we also show that bilateral midbrain transection could block i.p.-SCT-induced feeding reduction. Since we have previously shown the anorectic action of SCT via its effects on hypothalamic arcuate POMC neurons [16], we propose that SCT released from the gut may act in a paracrine fashion to activate SCTR in the vagal afferents, signalling to the NTS, which in turn communicates with the hypothalamus so as to modify appetite.

Apart from SCT, many other gut peptides have also been shown to influence appetite via the vagal afferent pathway. Including in the list are glucagon-like peptide 1 (GLP-1) [27], peptide YY [28], amylin [29], and cholecystokinin (CCK) [30], [31]. However, among these, GLP-1, CCK, and amylin all exemplify gut peptide that could inhibit appetite via vagal and non-vagal routes of communication with the brain [29], [32]. It was shown that reduction of food intake by i.p. injection, but not intravenous injection, of GLP-1 and CCK is abolished by capsaicin treatment or vagotomy [27], [32], whereas in the case of amylin, visceral afferents do not even seem to be involved in mediating the anorectic effect of i.p. administered amylin [29]. One possibility is that these peptides may have access to specific brain sites, including AP, where the BBB is incomplete as in the case of amylin. The other possibility is that these peptides could directly diffuse across the BBB as in the case of GLP-1 [33]. SCT have also been shown to have the ability to cross the blood-brain barrier [17]. However, as vagotomy and capsaicin treatment can both completely attenuate its anorectic effect, as well as its neuronal activating effect in AP, NTS and DMV, when i.p. was used as a route for SCT administration, therefore, modulation of food intake by i.p. SCT can be considered to neither involve a direct binding of the peptide to its receptor in specific brain regions lying outside the BBB, nor a direct diffusion of the peptide across the BBB. In conclusion, we believed that the satiety signal of peripheral SCT released from the gastrointestinal tract is sent via the vagus nerves to the brainstem and subsequently Arc, where it controls central expression of other regulatory peptides to regulate food intake.
